# Identification of a Chromosomal Integrated DNA Fragment Containing the *rmpA2* and *iucABCDiutA* Virulence Genes in Klebsiella pneumoniae

**DOI:** 10.1128/mSphere.01179-20

**Published:** 2020-12-23

**Authors:** Xuemei Yang, Lianwei Ye, Yi Li, Edward Wai-Chi Chan, Rong Zhang, Sheng Chen

**Affiliations:** a Department of Infectious Diseases and Public Health, Jockey Club College of Veterinary Medicine and Life Sciences, City University of Hong Kong, Hong Kong, China; b Department of Clinical Laboratory, Henan Provincial People’s Hospital, Zhengzhou, China; c State Key Laboratory of Chemical Biology and Drug Discovery, Department of Applied Biology and Chemical Technology, The Hong Kong Polytechnic University, Hung Hom, China; d Department of Clinical Laboratory, Second Affiliated Hospital of Zhejiang University, School of Medicine, Zhejiang, China; Antimicrobial Development Specialists, LLC

**Keywords:** *Klebsiella pneumoniae*, hypervirulence, virulence plasmid, chromosome fragment, transposition mechanisms

## Abstract

Klebsiella pneumoniae is increasingly being regarded as a reservoir of diverse antibiotic resistance genes and a pathogen that causes severe infections in both the hospital and the community. In this study, we performed molecular characterization of a carbapenem-resistant and highly virulent K. pneumoniae strain recovered from a hospital patient. The virulence-encoding genes *rmpA2* and *iucABCDiutA* were found to be located in the chromosome and are flanked by IS*26* elements. These chromosomally located virulence genes, if not incurring fitness costs, are expected to be much more stable than plasmid-located genes. Detailed analysis of the fragment in which these virulence genes are located showed that the fragment can readily form a circular intermediate that may promote integration of this virulence-encoding fragment into various plasmid backbones and other chromosomal regions. Findings in this work provide new insights into mechanisms of transmission of virulence-encoding genes in K. pneumoniae.

**IMPORTANCE** This study reported for the first time and characterized in detail the genetic features of a mobile virulence-encoding fragment located in the chromosome of a clinical virulent K. pneumoniae strain and revealed the occurrence of a transposition event mediated by IS*26*. This genetic structure could mediate the transposition of the virulence-encoding fragment into various plasmid backbones and chromosomes through formation of a circular intermediate. Therefore, findings in this work provide important insights into the transmission mechanisms of mobile virulence profiles in K. pneumoniae strains and lay the foundation for devising effective intervention approaches aimed at preventing the dissemination of these virulence-encoding elements.

## Abstract (precis)

This study reported for the first time and characterized in detail the genetic features of a mobile virulence-encoding fragment located in the chromosome of a clinical virulent K. pneumoniae strain and revealed the occurrence of a transposition event mediated by IS*26*. This genetic structure could mediate the transposition of the virulence-encoding fragment into various plasmid backbones and chromosomes through formation of a circular intermediate.

## INTRODUCTION

Klebsiella pneumoniae is a human commensal and opportunistic pathogen that can cause severe hospital-acquired infections, especially among patients with a compromised immune system (1). However, some of these “classic” K. pneumoniae (cKP) strains have acquired additional antibiotic resistance genes or virulence genes, and they have become resistant to multiple antibiotics or hypervirulent and cause infections that are hard to treat and often fatal (2, 3). In particular, hypervirulent K. pneumoniae (hvKP) strains can cause serious, life-threatening community-acquired infections among young and relatively healthy individuals (4, 5). K. pneumoniae employs a variety of virulence factors, such as capsule polysaccharide, enterobactin siderophore, lipopolysaccharide, and fimbriae, to evade and inhibit the host immune response, colonize the host, and obtain nutrition from the host (6). These virulence factors, which are chromosomally encoded, are common to all K. pneumoniae strains, including cKP and hvKP. Unlike cKP, hvKP mainly belonged to certain K1/K2 capsular serotypes and is associated with a large virulence plasmid that encodes the aerobactin iron uptake system and the regulator of the mucoid phenotype (7).

The accessory antimicrobial resistance and virulence determinants of K. pneumoniae are generally harbored by mobile genetic elements (MGEs), most typically plasmids and integrative and conjugative elements (ICEs), which can be horizontally transferred between K. pneumoniae strains (8, 9). These MGEs therefore play an important role in conferring multidrug resistance or hypervirulent phenotypes in K. pneumoniae. Lam et al. identified several broad families of virulence plasmids, among which the dominant types carried *iuc* and *iro* as well as *rmpA* determinants and include all previously described virulence plasmids of K. pneumoniae (10). These KpVP-1 and KpVP-2 virulence plasmids mainly belonged to the non-self-transmissible IncFIB_K_ type. In addition to the major virulence plasmids, integrated virulence-encoding plasmids that evolved from these well-studied plasmids and other plasmid backbones have been reported (2). In this study, we present a new mechanism underlying the dissemination of virulence elements in K. pneumoniae through formation of a circular intermediate; such a new virulence gene dissemination mechanism may speed up the rate by which clinical K. pneumoniae strains become hypervirulent.

## RESULTS AND DISCUSSION

### Phenotypic characterization.

K. pneumoniae strain 16HN-263, which was identified by the Vitek 2 system (bioMérieux, France) and confirmed by the matrix-assisted laser desorption ionization–time of flight mass spectrometry apparatus (Bruker, Germany), was recovered from a blood sample of a 53-year-old female patient in the intensive care unit of Henan Provincial People’s Hospital in 2016. This patient was diagnosed with a bacterial infection and was cured after antimicrobial treatment. Results of string tests indicated that strain 16HN-263 was string test negative. Antimicrobial susceptibility tests performed on strain 16HN-263 showed that it was resistant to all β-lactam antibiotics, amikacin, ciprofloxacin, and azithromycin but remained susceptible to polymyxin B and tigecycline (Table 1).

**TABLE 1:** MIC profiles of strain 16HN-263

Strain	MIC^*a*^ (μg ml^−1^)												
	ATM	CTX	CAZ	CMZ	FEP	IMP	MEM	EM	AMK	CIP	AZI	PB	TGC
16HN-263	>128	>128	>128	>128	>128	>128	>128	>128	>128	64	64	2	0.5
E. coli 25922	<0.5	<0.5	<0.5	<0.5	<0.5	<0.5	<0.5	<0.5	2	<0.5	2	<0.5	<0.5

a ATM, aztreonam; CTX, cefotaxime; CAZ, ceftazidime; CMZ, cefmetazole; FEP, cefepime; IMP, imipenem; MEM, meropenem; EM, ertapenem; AMK, amikacin; CIP, ciprofloxacin; AZI, azithromycin; PB, polymyxin B; TGC, tigecycline. All tests were performed in duplicate, and each test included three biological replicates.

### Genetic characterization.

The screening of virulence genes and carbapenemase gene *bla*_KPC_ showed that this strain harbored the *rmpA2* and *iucA* genes as well as the *bla*_KPC_ gene. CR-HvKP has been increasingly reported in China, and the evolution processes remain poorly understood (11). Strain 16HN-263 was subjected to whole-genome sequencing to further investigate the evolution of the virulence determinants. The genome size of strain 16HN-263 is 5,743,792 bp, including a 5.34-Mb chromosome (GenBank accession no. CP045263) and three plasmids with sizes of 129,252 bp (GenBank accession no. CP045264), 10,060 bp (GenBank accession no. CP045265), and 5,596 bp (GenBank accession no. CP045266). A total of 4,372 (∼75.5%) out of 5,790 predicted genes of the complete genome sequences were identified in the NCBI COG database. 16HN-263 was found to harbor one type II secretion system (T2SS)-encoding gene cluster, one type IV secretion system (T4SS)-encoding gene cluster, and two type VI secretion system (T6SS)-encoding gene clusters. This strain was found to belong to ST11 based on multilocus sequence typing (MLST) and KL47 serotype by Kaptive based on capsule synthesis loci (12). The chromosome was found to harbor several resistance genes, including *bla*_SHV-11_ (100% identity), *oqxAB* (100%), *fosA* (100%), *sul1* (100%), *aadA2* (99.75%), and *mph*(E) (100%). The 129,252-bp plasmid was found to belong to the IncFII_33/IncR type with a GC content of 54.46% and was comprised of 175 predicted protein-coding genes. This plasmid harbored the carbapenemase gene *bla*_KPC-2_ (100% identity) and was designated p16HN-263_KPC. Being highly similar (99% coverage and 99% identity) to plasmid p69-2 (GenBank accession no. CP025458.1) and (91% coverage and 99% identity) to plasmid pCR-HvKP4-KPC (GenBank accession no. CP040541.1), plasmid p16HN-263_KPC also harbored *bla*_TEM-1_ (100%), *bla*_CTX-M-65_ (100%), *bla*_SHV-12_ (100%), *catA2* (96.11%), *fosA3* (100%), and *rmtB* (100%) resistance genes (see Fig. S1a in the supplemental material). This kind of *bla*_KPC-2_-bearing plasmid is widespread in K. pneumoniae strains isolated from China (11, 13). The others were two small ColRNAI plasmids, designated p16HN-263_2 and p16HN-263_3. These two small plasmids were found to be harbored by K. pneumoniae strains characterized by our laboratory previously (14). The plasmidome of strain 16HN-263 has been aligned to our previously characterized CR-HvKP strains. Plasmid p16HN-263_2 lacked one gene compared to plasmid p4, and plasmid p16HN-263_3 was exactly the same as p5 of these CR-HvKP strains (Fig. S1b and c). The plasmidome analysis indicated that the three plasmids of strain 16HN-263 evolved from our previously characterized CR-HvKP strains (14). This strain did not harbor a pLVPK-like virulence plasmid, as reported in other K. pneumoniae strains, which carried virulence factors such as the *rmpA2* and *iucA* genes.

10.1128/mSphere.01179-20.1 FIG S1 Plasmidome analysis of strain 16HN-263 compared to CR-HvKP strains. (A) Alignment of plasmid p16HN-263_KPC with the plasmids pCR-HvKP1-KPC, pCR-HvKP4-KPC, pCR-HvKP5-KPC, and p69-2. (B) Alignment of plasmid p16HN-263_2 with the plasmids pCR-HvKP1-p4, pCR-HvKP4-p4, and pCR-HvKP5-p4. (C) Alignment of plasmid p16HN-263_3 with the plasmids pCR-HvKP1-p5, pCR-HvKP4-p5, and pCR-HvKP5-p5. Download FIG S1, TIF file, 2.0 MB. Copyright © 2020 Yang et al. 2020 Yang et al. This content is distributed under the terms of the Creative Commons Attribution 4.0 International license.

**FIG 1:**
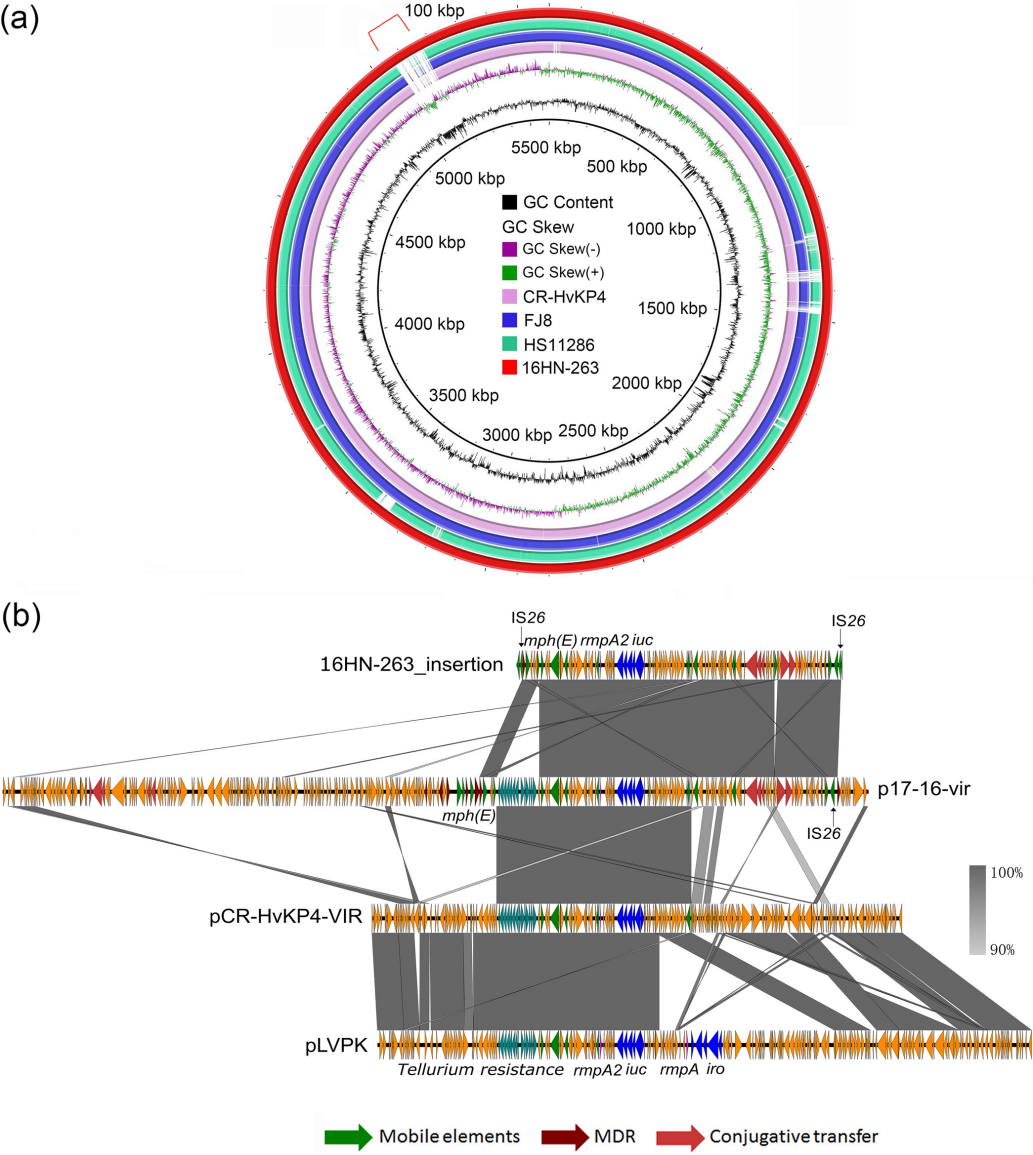
Detailed genetic features of a virulence-encoding region in the chromosome of strain 16HN-263. (a) Sequence alignment of four complete K. pneumoniae chromosomal sequences, with the chromosome of 16HN-263 being used as a reference. Alignment with the chromosomal sequence of strain HS11286 (GenBank accession no. CP003200) and those of our previously reported strains, CR-HvKP4 and FJ8, indicates that a 100-kbp segment was inserted into the chromosome of strain 16HN-263. (b) Alignment of the 107,648-bp region in the chromosome of strain 16HN-263 with the plasmids p17-16-vir (GenBank accession no. MK191024.1), pCR-HvKP4_VIR (GenBank accession no. CP040540.1), and pLVPK (GenBank accession no. AY378100.1).

BLASTN against the virulence genes database (http://bigsdb.pasteur.fr/klebsiella/klebsiella.html) showed that strain 16HN-263 harbored a number of virulence genes, including type 3 fimbria-encoding genes *mrkABCDFHIJ*, yersiniabactin-encoding genes, regulator of mucoid phenotype gene *rmpA2*, and aerobactin-encoding genes *iucABCDiutA*, thereby confirming the screening results. The aerobactin lineage was predicted as *iuc1*, and *rmpA2* was determined as variant *rmpA2_8* according to Kleborate. Interestingly, the *rmpA2* and *iucABCDiutA* genes were found to be located in the chromosome of strain 16HN-263 rather than in a plasmid. Chromosomal *rmpA* has been described in K. pneumoniae strain NTUH-K2044 previously, which was located in ICEKp1 together with the *iroBCDN* gene cluster (15, 16). Recent studies suggested that *rmpA* mainly activates the expression of *rmpD* and *rmpC* to produce the hypermucoviscous phenotype and stimulate capsular expression, respectively (17, 18), while strain 16HN-263 did not harbor the newly identified *rmpC* and *rmpD* genes, which may result in the negative string test. The virulence-associated genes of strain 16HN-263 were located on a 100-kbp insertion fragment flanked by IS*26* mobile elements (Tn*7074*) (19) (Fig. 1a). To confirm that Tn*7074* is truly integrated into the chromosome, nanopore reads were aligned to the assembled chromosome. A total of 13 nanopore reads were found to span the upstream integration site and 8 reads to span the downstream integration site (Fig. S2a). Furthermore, we designed primers to amplify across the ends of the integration site. The obtained PCR products were consistent with the expected size (Fig. S2b). The exact size of this insertion fragment was found to be 107,648 bp with a corresponding G+C content of 48.2% and comprised 120 predicted open reading frames. The genes located in this fragment included the macrolide resistance gene *mph*(E), virulence-associated genes *rmpA2* and *iucABCDiutA*, and the IncF plasmid-based *tra* genes. BLAST against the NCBI database using an algorithm of BLASTn showed that this fragment exhibited the highest level of sequence identity to plasmid p17-16-vir (GenBank accession no. MK191024.1), with coverage of 98% and identity of 100%, which was also harbored by a clinical K. pneumoniae strain isolated in Zhengzhou. Alignment of Tn*7074* with p17-16-vir and the virulence plasmids pCR-HvKP4_VIR (GenBank accession no. CP040540.1) and pLVPK (GenBank accession no. AY378100) indicated that this fragment originated from a fusion of a pCR-HvKP4_VIR-like virulence plasmid and an IncF-type resistance plasmid (Fig. 1b).

10.1128/mSphere.01179-20.2 FIG S2 Confirmation of the integration of a 100-kbp virulence and resistance encoding region into the chromosome. (A) Alignment of nanopore reads (SRR12072214) to chromosome assembly of 16HN-263 (GenBank accession no. CP045263.1). The alignment was generated by BWA-MEM and viewed by IGV. (B) Gel electrophoresis of PCR amplicons across both ends of the integration sites. “Up” depicts the PCR product of the upstream integration site; “Down” depicts the PCR product of the downstream integration site. Download FIG S2, TIF file, 0.2 MB. Copyright © 2020 Yang et al. 2020 Yang et al. This content is distributed under the terms of the Creative Commons Attribution 4.0 International license.

### Transmission mechanism.

**FIG 2:**
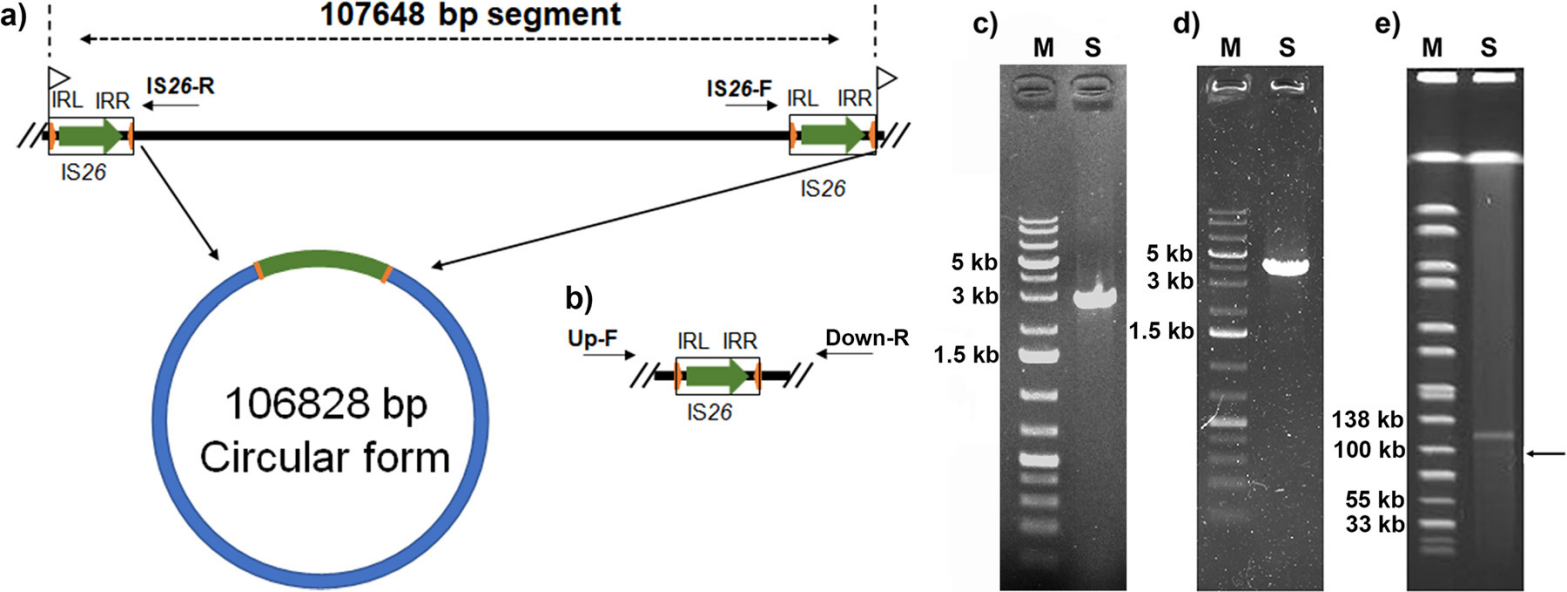
Formation of a circular intermediate by circularization of the insertion segment in the chromosome of strain 16HN-263. (a) Genetic structure of the transposon and its circular intermediate. IS*26* elements are shown as open boxes. Green arrows indicate the position and orientation of *tnp26*. Orange arrows refer to the left and right terminal inverted repeats (IRL and IRR) of the IS*26* element. Flags indicate the position and direction of the identified target site duplication (TSD). (b) Genetic structure of the chromosomal fragment after excision of the transposon. (c) Gel electrophoresis of PCR amplicons corresponding to the circular intermediate generated using the reverse primers IS*26*-F and IS*26*-R. S depicts the PCR product. (d) Gel electrophoresis of PCR amplicons corresponding to the excision of Tn*7074* using the primers Up-F and Up-R. S depicts the PCR product. (e) S1-PFGE profile of strain 16HN-263. S indicates strain 16HN-263.

The inserted segment was found to be flanked by two IS*26* elements. An 8-bp target site duplication (TSD) (5′-CGAAGAAC-3′) was identified and flanked the two IS*26* elements (Fig. 2a). A common mechanism by which a transposon is disseminated to other genetic fragments is to form active transposition intermediates (20). A set of outward-facing primers targeting the insertion sequence was used to investigate the potential of this segment to circularize (Fig. 2a). As a result, a PCR fragment with a size of about 3 kb was obtained and was found to be consistent with the expected size, showing that the chromosome inserted segment could be circularized (Fig. 2c). The DNA sequences of the obtained PCR fragment matched the genome assembly around the two IS*26* elements. The two IS*26* elements were identical to the reference without any mutation. The amplified sequences spliced the two ends of the fragment through the homologous IS*26* elements, resulting in only one IS*26* element in the circular form (Fig. 2a). IS*26* plays an important role in disseminating antibiotic resistance genes, forming regions containing antibiotic resistance genes that were flanked by two IS*26*s. In most cases, the IS*26*s are in a direct orientation, and either upstream or downstream IS*26* could mediate the cointegration into different sites (21). A single IS*26* element could mediate the movement of the translocatable unit (TU), which preferentially inserts adjacent to another IS*26* (22). Plasmid p17-16-vir was found to carry an IS*26* element at the downstream end of the region homologous to the insertion fragment, although there is no IS*26* near the upstream end of the region (Fig. 1b). This provided further support for the hypothesis that the inserted fragment originated from a plasmid like p17-16-vir. The excision of Tn*7074* was further determined by PCR using primers Up-F and Down-R (Fig. 2b). The PCR fragment matched the genome assembly surrounding the two IS*26* elements with excision of Tn*7074* and retaining one IS*26* (Fig. 2d). This finding was further confirmed by the S1 nuclease pulsed-field gel electrophoresis (S1-PFGE) profile of strain 16HN-263, which depicted the presence of a weak band of around 100 kb in size under the *bla*_KPC-2_-bearing plasmid (Fig. 2e). There were no nanopore sequences that span the 5′ and 3′ ends of the circularized composite Tn*7074* or the excised chromosome. Thus, excision frequency failed to be calculated based on Nanopore reads. While long reads that span each end of Tn*7074* and the chromosomal integration site were found (Fig. S2), this may be due to the low sequencing coverage and low excision frequency. The transmission process could be explained by the mechanism of intermolecular replicative transposition, which results in the formation of a cointegrate of the donor and recipient molecules, with a directly repeated copy of IS*26* at each junction (8). In summary, the virulence-encoding DNA fragment was mainly integrated on the chromosome of 16HN-263 with the dynamic presence of a circular intermediate to make the transmission of the fragment. Conjugation was performed using strain 16HN-263 as the donor and rifamycin-resistant Escherichia coli strain EC600 as the recipient, as previously described (2). However, even though multiple attempts were tried, we were still unable to get transconjugants. This may be due to the incomplete conjugation gene clusters in this fragment.

### Virulence potential of strain 16HN-263.

**FIG 3:**
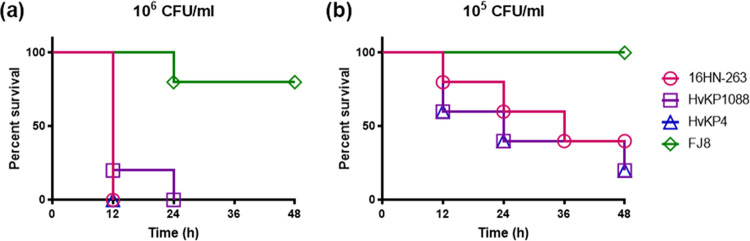
Virulence level of different bacterial strains as depicted in a wax worm (Galleria mellonella) larva infection model. (a and b) Survival of wax worm (*n* = 10) infected by 1 × 10^6^ CFU/ml (a) and 1 × 10^5^ CFU/ml (b) of each K. pneumoniae strain at 48 h. The test strains included K. pneumoniae strain 16HN-263, ST11 CR-HvKP strain CR-HvKP4 (hypervirulence control), ST23 K1 strain HvKP1088 (hypervirulent K. pneumoniae control), and the classic CRKP strain FJ8 (low-virulence control). A log rank (Mantel-Cox) test was performed for the indicated curves. A significant difference (*P =* 0.0027 [a] and *P *= 0.0494 [b]) was observed between 16HN-263 and FJ8, while no significant difference was observed between 16HN-263 and CR-HvKP4 or HvKP1088 (*P* > 0.9999 and *P *= 0.5417 between 16HN-263 and CR-HvKP4 in panels a and b, respectively; *P *= 0.3173 and *P* = 0.5417 between 16HN-263 and HvKP1088 in panels a and b, respectively).

The virulence level of strain 16HN-263 was tested in wax worm (Galleria mellonella) larvae. Upon being infected for 48 h with an inoculum of 1 × 10^6^ CFU/ml of these strains, survival of G. mellonella was 0% for 16HN-263 and the hypervirulence control strain CR-HvKP4 and HvKP1088, but 80% survival was recorded for the low-virulence control strain FJ8 (Fig. 3a). Infection by a lower bacterial dose, namely, a concentration of 1 × 10^5^ CFU/ml, resulted in 20%, 20%, and 40% survival for CR-HvKP4, HvKP1088, and 16HN-263, respectively, but a 100% survival rate was recorded for FJ8 (Fig. 3b). A log rank (Mantel-Cox) test was performed for the indicated curves. A significant difference (*P *= 0.0027 and *P *= 0.0494 in both doses, respectively) was observed between 16HN-263 and FJ8, while no significant difference was observed between 16HN-263 and CR-Hvkp4 or HvKP1088 at both doses (*P * > 0.9999 and *P *= 0.5417 between 16HN-263 and CR-Hvkp4, respectively; *P *= 0.3173 and *P *= 0.5417 between 16HN-263 and HvKP1088, respectively). These data indicated that in the wax worm infection model, 16HN-263 exhibited a virulence level less than that of the ST11 hypervirulent K. pneumoniae control strain, CR-HvKP4, and the ST23 hypervirulent K. pneumoniae control strain, HvKP1088, but higher than that of the classic ST11 strain FJ8, suggesting that strain 16HN-263 was phenotypically highly virulent compared to classic ST11 strain FJ8.

### Conclusions.

In conclusion, this study reported, for the first time, and characterized in detail the genetic features of a mobile virulence-encoding fragment located in the chromosome of a clinical virulent K. pneumoniae strain and revealed the occurrence of a transposition event mediated by IS*26*. This genetic structure could mediate the transposition of the virulence-encoding fragment into various plasmid backbones and chromosomes through the formation of a circular intermediate. Chromosomally located virulence genes are less likely to be lost during cell division and more stable than plasmid-located genes if not incurring fitness costs. Therefore, findings in this work provide important insights into the transmission mechanisms of mobile virulence profiles in K. pneumoniae strains and lay the foundation for devising effective intervention approaches aimed at preventing dissemination of these virulence-encoding elements.

## MATERIALS AND METHODS

### Bacterial strains and identification.

*Klebsiella* strain 16HN-263 was obtained from a patient in Henan Provincial People’s Hospital (Zhengzhou, China) in 2016. Virulence genes (*rmpA*, *rmpA2*, *iucA*, and *iroN*) and carbapenemase gene *bla*_KPC_, harbored by this strain, were screened by PCR using primers as described previously (11). A string test was performed on blood agar as previously described (11).

### Antibiotic susceptibility test.

Antimicrobial susceptibility of strain 16HN-263 was determined by the microdilution method according to the guidelines recommended by the Clinical and Laboratory Standards Institute (23). E. coli strain 25922 served as a quality control strain for susceptibility testing. Antimicrobial agents tested included aztreonam, cefotaxime, ceftazidime, cefmetazole, cefepime, meropenem, imipenem, ertapenem, ciprofloxacin, amikacin, azithromycin, polymyxin B, and tigecycline. All tests were performed in duplicate, and each test included three biological replicates. The breakpoints of antimicrobial agents tested, except tigecycline, were interpreted according to the CLSI guidelines. The breakpoint of tigecycline was interpreted according to the European Committee on Antimicrobial Susceptibility Testing (EUCAST) guidelines (https://eucast.org/clinical_breakpoints/).

### DNA sequencing and bioinformatics analysis.

Genomic DNA was extracted using the Genomic purification kit for bacteria (Qiagen, Germany). The extracted DNA was then subjected to library preparation by a NEBNext Ultra II DNA library prep kit for Illumina (New England Biolabs, USA) and sequenced via the 150-bp paired-end Illumina NextSeq 500 platform (Illumina, San Diego, CA). Genomic DNA was also subjected to the long-read Oxford Nanopore Technologies MinION platform after treatment with a supplementary sequencing kit (Nanopore, Oxford, United Kingdom). Both short and long reads were *de novo* hybrid assembled using Unicycler v0.4.7 (24). Assembled genome sequences were annotated with RAST v2.0 (25). Clusters of Orthologous Groups of proteins (COGs) were predicted using rpsblast against the NCBI COG database (26). Multilocus sequence typing (MLST) was determined by the Kleborate software based on the types of genetic variations in the seven housekeeping genes (https://github.com/katholt/Kleborate). Capsular typing on the assembled sequences was performed using Kaptive (12). Virulence genes were identified by searching against the BIGSdb *Klebsiella* genome database (http://bigsdb.pasteur.fr/klebsiella/klebsiella.html). The BLASTn command lines, with an 80% coverage and identity cutoff, were used to map genome sequences against antibiotic resistance genes, insertion sequences, and plasmid replicons. The resistance gene and plasmid replicon databases were obtained from the Center for Genomic Epidemiology (http://www.genomicepidemiology.org/). The IncFII alleles were determined by Plasmid MLST schemes (27). Alignment of plasmids with similar structures was generated by BLAST Ring Image Generator (BRIG) version 0.95.22 (28) and Easyfig_win_2.1 (29).

### Confirmation of the integration of a 100-kbp virulence- and resistance-encoding region into the chromosome.

A fragment containing virulence genes *rmpA2* and *iuc*, macrolide resistance gene *mph*(E), *tra* genes, and mobile elements was found to be inserted into the chromosome of strain 16HN-263. To confirm that the 100-kbp region is truly integrated into the chromosome, Nanopore reads were aligned to the assembled chromosome by Burrows-Wheeler Aligner’s BWA-MEM algorithm (30). The resulting alignment was then converted to bam format and sorted by SAMtools (31). The aligned result was viewed by Integrative Genomics Viewer (IGV) (32). We then designed two pairs of primers to amplify across both ends of the integration sites. The upstream integration site was amplified using primers (Up-F, TCGGTCATAACCTGGAGATC; Up-R, GCATTCCACGCTCTTCGAAA) with an expected size of 3,932 bp. The downstream integration site was amplified using primers (Down-F, GAGCGTTCTGGACTGTTTG; Down-R, TGTGCTTCAGCAAGTGATTG) with an expected size of 3,512 bp.

### Detection of circular intermediates.

A set of outward-facing primers (forward, GCATTCCACGCTCTTCGAAA; reverse, TGTGCTTCAGCAAGTGATTG) targeting the insertion sequence (Tn*7074*) was designed to investigate the potential of this segment to circularize. The expected size of the PCR fragment including one of the IS*26* mobile elements based on the genome assembly was as large as 2,825 bp. The PCR products were sequenced by the Beijing Genomics Institute (BGI) and mapped to the chromosome assembly to determine the complete map of the circular intermediate. S1 nuclease pulsed-field gel electrophoresis (S1-PFGE) was performed as described previously (33). The excision of Tn*7074* from the chromosome was determined by PCR using primers Up-F and Down-R. The PCR products were sequenced by BGI and mapped to the chromosome assembly. The presence of Nanopore long reads covering the excised Tn*7074* was investigated. Conjugation has been performed using strain 16HN-263 as the donor and rifamycin-resistant E. coli strain EC600 as the recipient as previously described (2). The transconjugants were screened by China Blue agar plates containing 8 μg/ml azithromycin and 600 μg/ml rifamycin.

### Wax moth larva model.

The virulence level of the test strains was determined in wax moth (Galleria mellonella) larvae weighing about 300 mg (purchased from Tianjin Huiyude Biotech Company, Tianjin, China). Briefly, overnight cultures were washed with phosphate-buffered saline (PBS) and further adjusted with PBS to concentrations of 1 × 10^4^ CFU/ml, 1 × 10^5^ CFU/ml, 1 × 10^6^ CFU/ml, and 1 × 10^7^ CFU/ml. Ten G. mellonella larvae in each group were infected with the bacteria in 10-μl inocula and incubated at 37°C. The survival rate of G. mellonella was recorded for 48 h (34). A ST11 CR-HvKP strain CR-HvKP4 and a typical ST23 K1 HvKP strain HvKP1088, which were demonstrated to be hypervirulent in our previous reports, were used as hypervirulence controls (3, 11). A classic ST11 CRKP strain FJ8, which was reported in our previous study, was used as a low-virulence control. Animal experiments were repeated at least twice to assess the consistency of the data. Survival curves were generated using GraphPad Prism 7.00. Statistical analysis was performed using the log rank (Mantel-Cox) test recommended by Prism 7.00.

### Data availability.

Complete sequences of the chromosome of strain 16HN-263, plasmid p16HN-263_KPC, p16HN-263_2, and p16HN-263_3 have been deposited in the GenBank database under accession numbers CP045263 to CP045266, respectively. Illumina and Nanopore read data have been deposited in the GenBank database under accession numbers SRR12072215 and SRR12072214.
