# Supplementary figures and images for: Identification of a Chromosomal Integrated DNA Fragment Containing the rmpA2 and iucABCDiutA Virulence Genes in Klebsiella pneumoniae
Source: mSphere. 2020 Dec 23;5(6):e01179-20. doi: 10.1128/mSphere.01179-20 (PMC7763553; doi:10.1128/mSphere.01179-20)

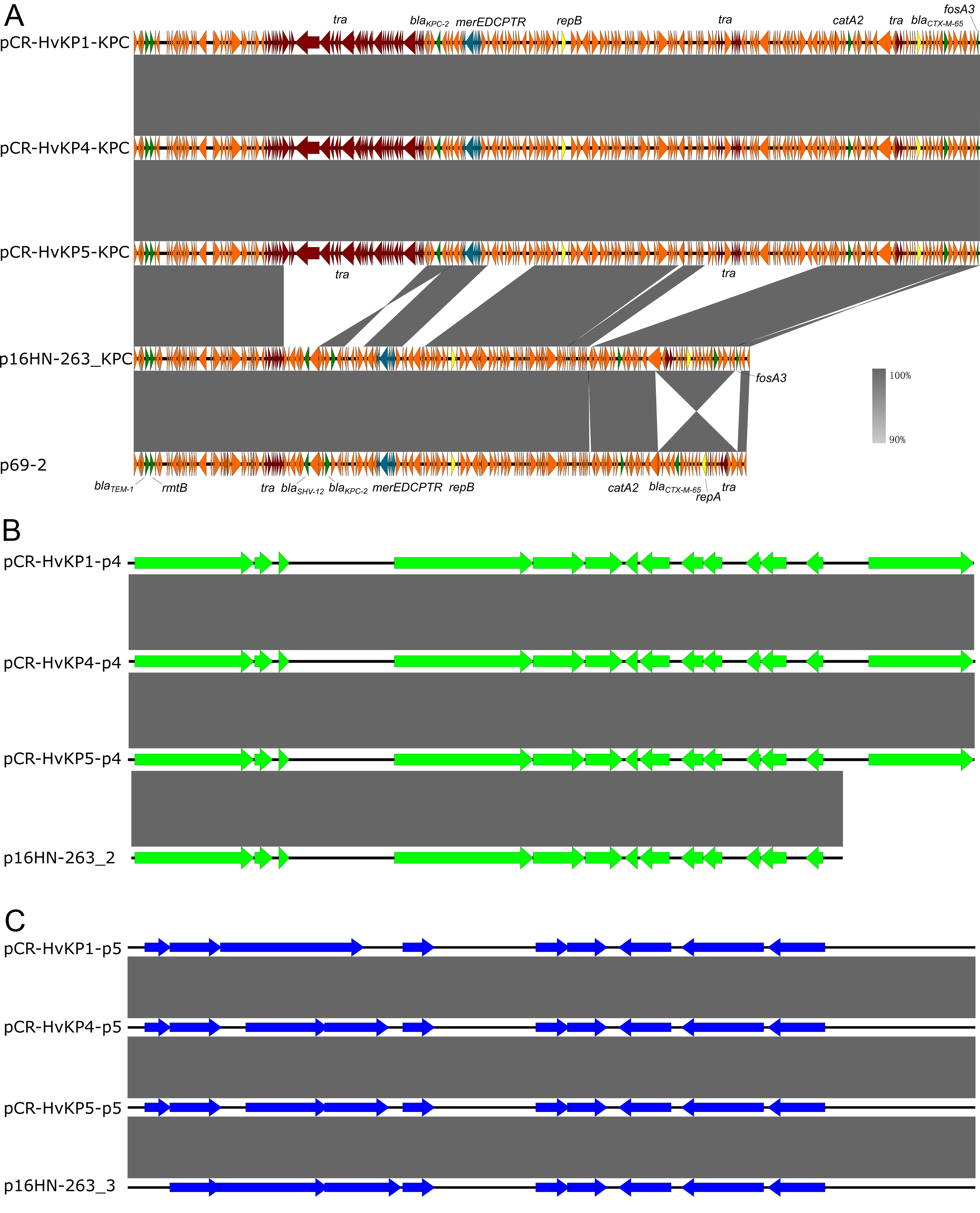

Supplement: FIG S1 [file mSphere.01179-20-sf001.tif]

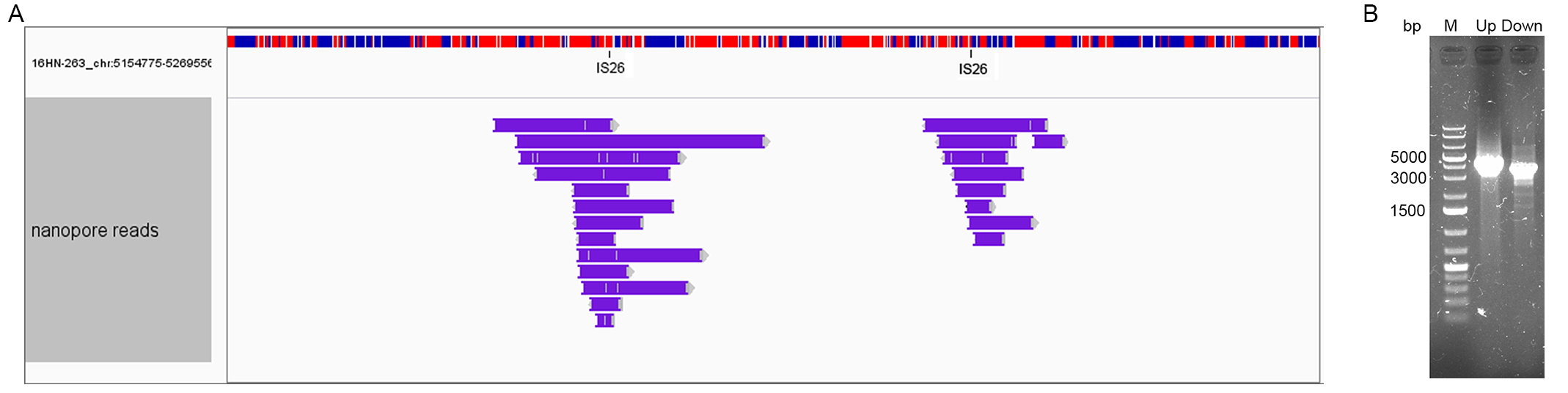

Supplement: FIG S2 [file mSphere.01179-20-sf002.tif]
